# Development of an Aptamer-Based Surface Plasmon Resonance Biosensor for Detecting Chloramphenicol in Milk

**DOI:** 10.3390/bios15110706

**Published:** 2025-10-22

**Authors:** Minyu Qi, Ningqi Xia, Xiying Wang, Xiaofei Wang, Hao Chen, Diya Lv, Yan Cao

**Affiliations:** 1 Department of Biochemical Pharmacy, College of Pharmacy, Naval Medical University, Shanghai 200433, China; jstzqi@163.com (M.Q.); xianingqiqi@163.com (N.X.);; 2 Suzhou Innovation Center of Shanghai University, Suzhou 215127, China; 3 Center for Instrumental Analysis, College of Pharmacy, Naval Medical University, Shanghai 200433, China; 4 Shanghai Key Laboratory for Pharmaceutical Metabolite Research, College of Pharmacy, Naval Medical University, Shanghai 200433, China

**Keywords:** surface plasmon resonance, aptamer, immobilization strategy, chloramphenicol

## Abstract

Surface plasmon resonance (SPR) biosensors have been applied in various fields with the advantages of being label-free, having high specificity, having high sensitivity, and providing real-time monitoring. With the gradual improvement of SPR technology, SPR biosensors have been used for the detection of macromolecules such as proteins, peptides, and nucleic acids. Antibodies are generally used as the recognition component of SPR biosensors due to the high specificity of antibody–antigen binding. Recently, aptamers have become new choices instead of antibodies for their characteristic of high specificity with target molecules, high stability of chemical synthesis, convenience in storage, and ease of labeling. In this study, an aptamer-based SPR biosensor for chloramphenicol (CAP) detection was established through optimizing the conditions of CAP aptamer immobilization and analysis procedure, including biosensor type, signal enhancement, running buffer, sample diluent, and dissociation time. The results suggested that the optimal immobilization strategy of aptamers on the SPR biosensor was indirect immobilization based on the CM5 chip. The aptamer-based SPR biosensor had good specificity for CAP and could be used to detect CAP in real samples such as milk. Therefore, SPR biosensors have great application prospects in the food safety field, and aptamers deserve further study to improve the performance of the biosensor.

## 1. Introduction

Chloramphenicol (CAP) is a broad-spectrum antibiotic that can treat infections caused by Gram-positive, Gram-negative, and anaerobic bacteria and has been widely used in human medicine, aquaculture, and food-animal agriculture [1]. CAP can cause serious adverse reactions in humans, such as bone marrow depression, fatal aplastic anemia, leukemia, and potentially genotoxic carcinogenicity. Accordingly, the usage of CAP in food-producing animals has been banned in the European Union, China, the USA, Canada, Australia, and Japan [2,3], which means that CAP must not be detected in food. However, due to the dual advantages of low production cost and high stability, chloramphenicol is still widely used in many developing countries [3]. The illegal use of CAP to treat cows through intramuscular injection or the intramammary route can lead to the presence of CAP in milk, and the further transfer of CAP in milk to various dairy products increases the possibility of adverse effects [4]. Traditional CAP detection methods have the disadvantages of high cost and time-consuming and cumbersome processing steps [5]. Thus, it is necessary to develop a CAP detection method with the characteristics of real-time, good stability, and easy-to-operate to satisfy more needs of CAP detection in milk.

Surface plasmon resonance (SPR) biosensors convert light signals into response values based on optical principles and can detect intermolecular interactions of biomolecules. It has been applied in clinical analysis, environmental monitoring, and food analysis with characteristics of label-free, high specificity, high sensitivity, and real-time monitoring [6,7,8]. Xiao et al. [9] developed a portable smartphone-based imaging SPR biosensor for the quantitative analysis of total hazelnut protein in five different plant-based milks and showed the sensitivity of direct SPR immunoassay was comparable to or better than commercially available hazelnut immunoassays. He et al. [10] performed a detection of 3-nitrotyrosine in human urine based on anti-3-nitrotyrosine antibody using an SPR sensor, compared with enzyme-linked immunosorbent assay, and demonstrated high sensitivity, selectivity, and good stability of the SPR sensor. Guo et al. [11] developed a direct SPR biosensor with anti-triazophos monoclonal antibodies immobilized on the sensor chip to detect triazophos in the spiked environmental water, agricultural products, and real-life samples. For the high specificity of antibody–antigen binding, antibodies are generally used as biological recognition elements to improve the sensitivity and accuracy of SPR biosensors. However, the hidden huge dangers of antibodies have been found recently. A considerable amount of published research based on antibodies cannot be reproduced by antibodies from the same antibody manufacturer in other experiments because of the inaccurate information of antibodies provided by manufacturers and the instability of antibodies during storage and transport [12,13].

In order to improve the stability and reproducibility of SPR biosensors, aptamers are another option besides antibodies. Aptamers are short single-stranded nucleic acid sequences that can fold into diverse three-dimensional structures [14,15]. Aptamers are also called “chemical antibodies” because the conformational recognition of aptamers and targets is similar to that of antibodies-antigens, and they have the characteristics of high affinity and specificity [16]. Importantly, aptamers have several advantages over antibodies: first, aptamers are produced by chemical synthesis, which has largely avoided batch-to-batch variation and guaranteed high stability, and the reproducibility of the experiment can benefit from it [17]. Second, chemical modifications can be freely added during synthesis to enhance specificity and stability, which increases the flexibility of experiments [15,18,19]. Due to the above advantages, aptamers are gradually being used as recognition elements of biosensors besides antibodies.

Up to now, aptamer-based SPR biosensors have been applied in various areas, including food analysis and clinical diagnosis. Wu et al. [20] developed an aptamer-based SPR sensor for detecting aflatoxin and confirmed the advantages of this sensor of short detection time, high sensitivity, and high specificity compared to the previously reported methods. Lee et al. [21] applied an SPR biosensor with a mixed aptamer-modified chip to detect two cardiac disease protein biomarkers, NT-proBNP and TNF-α, simultaneously. The SPR biosensor was demonstrated to be capable of detecting human serum samples and had better sensitivity than the ELISA method. Zhang et al. [22] developed an angle-scanning SPR biosensor for glycated hemoglobin detection and demonstrated the low limit of detection and wide detection range of this sensor. The three-dimensional structure of aptamers requires space on the chip surface to expand. Therefore, the density of the recognition element immobilized on the sensor surface is important. The density cannot be too high due to steric hindrance, but low density may reduce the signal value, which is unfavorable for detection. Simon et al. [23] confirmed that the surface density of aptamers indeed affected the target binding efficiency by SPR imaging technology through human immunoglobulin E, and the optimal surface densities were related to the size of the target. However, the responses of small molecules are much lower than that of macromolecules in SPR detecting; thus, it requires higher aptamer coupling levels and more complicated analysis conditions, which hinders the application of aptamer-based SPR sensors to small molecule detection. Blidar et al. [24] developed an aptamer-SPR sensor for ampicillin detection, based on enhanced immobilization of the specific aptamer by a potential pulsing electrochemical method on the gold chip. This aptasensor was able to detect ampicillin in the linear range of 2.5–1000 μM with a limit of detection (LOD) of 1 μM. Kang et al. [25] fabricated an aptamer-based SPR sensor and applied it for the direct detection of oxytetracycline. This aptamer SPR sensor had a detection range of 1.56 nM to 3.2 µM and an LOD of 3.6 nM.

In this study, we aimed to establish an aptamer-based surface plasmon resonance biosensor through optimizing the conditions of aptamer immobilization and analysis process and to apply the SPR sensor to detecting CAP. The effects of chip type, signal enhancement, running buffer and sample diluent, and dissociation time on the aptamer biosensor were studied, and the optimal scheme was determined. Finally, the proposed aptamer biosensor can detect CAP, which was added to milk. The study proves that aptamer-based SPR biosensors can be applied to detecting small molecules and have the potential to become one of the mainstream detection tools of milk and more other food samples with the intrinsic advantages of the aptamer in the future.

## 2. Materials and Methods

### 2.1. Drugs and Reagents

Chloramphenicol (CAP), thiamphenicol (THP), florfenicol (FF), and cefuroxime (CXM) were purchased from Efebio (Shanghai, China) with a purity of 98%. Streptavidin (SA) was provided by Absin (Shanghai, China). NaCl, MgCl_2_, KCl, CaCl_2_, 1 M Tris-HCl (pH 7.4–7.6) were purchased from Sangon Biotech Co., Ltd. (Shanghai, China). All DNA aptamers for CAP (HPLC-purified lyophilized powder) were synthesized by Sangon Biotech Co., Ltd. (Shanghai, China). The core sequence of the CAP aptamer was selected from reference 26 (clone7) and reference 27 (LR20), with partial modifications as shown in Table 1 [26,27]. CM5 sensor chips, SA sensor chips, 1-(3-Dimethylaminopropyl)-3-ethylcarbodiimide (EDC), N-Hydroxy succinimide (NHS), ethanolamine, phosphate-buffered saline (PBS), HBS-EP+ buffer were purchased from GE Healthcare (Waukesha, WI, USA). Purified water was acquired from a Milli-Q system (Millipore, Bedford, MA, USA).

### 2.2. Sample Preparation and Aptamer Pretreatment

CAP, THP, FF, and CXM were first dissolved to 5 mM in purified water. Sample dilution buffers A, B, and C were prepared according to Table 2. All buffers were filtered by a mixed cellulose filter membrane (0.22 μm, 25 mm) after preparation.

All aptamers were dissolved to 1 mM using buffer Buf1 and then diluted to the required concentrations. The diluted aptamer solution was heated to 95 °C for 5 min and annealed by cooling to 25 °C within 30 min to fold into the correct structure in the PCR apparatus (LongGene, Hangzhou, Zhejiang, China).

### 2.3. Strategy of Amino Coupling on CM5 Chip

#### 2.3.1. Coupling of Apt1 on CM5 Chip

All SPR biosensor analyses were performed on a Biacore T200 system (GE Healthcare, Chicago, IL, USA). The flow rate of running buffer (HBS-EP+ buffer) was 10 μL/min, and the system temperature was 25 °C. Flow cell (FC) 1 of a CM5 chip was set as the reference cell, and FC 2 as the detection cell. The optimal coupling conditions of aptamer were determined by physical absorption progress using 10 mM sodium acetate buffers (pH 4.0, 4.5, 5.0, 5.5) to dilute the Apt1 to 10 μM.

The Apt1 was immobilized on the CM5 chip through an amino coupling reaction. Briefly, the FC 2 of the SPR sensor chip was activated with a mixed solution of EDC and NHS for 420 s at a flow rate of 10 μL/min. Then, the prepared Apt1 was injected for 600 s, enabling the coupling process. After that, the activation sites without Apt1 coupled were blocked with ethanolamine for 420 s.

#### 2.3.2. Activity of Apt1-Based SPR Biosensor

The running buffer was Buf1, and the 5 mM CAP was diluted successively to 16, 8, 4, 2, 1, 0.5, 0.25, and 0.125 μM with Buf1. Samples were injected into FC 1 and FC 2 in the order of concentration from low to high. All samples were injected on the sensor surface for 120 s and dissociated for 180 s at a flow rate of 20 μL/min.

### 2.4. Strategy of Biotin-Avidin Coupling on SA Chip

#### 2.4.1. Immobilization of Apt2 and Apt3 on SA Chip

FC 1 and FC 3 of an SA chip were set as reference cells, and FC 2 and FC 4 as detection cells. Apt2 and Apt3 were, respectively, immobilized on FC 2 and FC 4 by flowing through the flow cell at a flow rate of 10 μL/min, and the running buffer was Buf1. The concentration of both aptamers was 10 μM, and the injection time was 600 s.

#### 2.4.2. Activity of SPR Biosensor with SA Chip

The 5 mM CAP was diluted sequentially with Buf1 to 1.6, 0.8, 0.4, 0.2, 0.1, 0.05, and 0.025 μM. All samples were injected into FC 1 and FC 2 in the order of concentration from low to high for 120 s, and the dissociation time was 240 s. The flow rate was 20 μL/min. Similarly, the 5 mM CAP was diluted successively to 16, 8, 4, 2, 1, 0.5, and 0.25 μM with Buf1, and the samples were injected into FC 3 and FC 4 under the same analysis conditions. The equilibrium dissociation constant (K_D_) was determined by Biacore T200 Evaluation Software (version 2.0).

### 2.5. Strategy of Biotin-Avidin Coupling on CM5 Chip

#### 2.5.1. Immobilization of SA on CM5 Chip

SA was diluted with 10 mM sodium acetate buffer (pH 4.0, 4.5, 5.0, 5.5) to 100 μg/mL to determine the optimal coupling condition by physical absorption progress. SA was immobilized on FC 1 and FC 2 of a CM5 chip with HBS-EP+ running buffer using an amino coupling reaction as described in Section 2.3.1.

#### 2.5.2. Immobilization of Apt2 on CM5 Chip

Apt2 was diluted to 10 μM with Buf1 and then injected into FC 2 for 600 s at a flow rate of 10 μL/min with the PBS running buffer.

#### 2.5.3. Activity of CM5 Chip with Apt2 and LOD

The 5 mM CAP was diluted sequentially with Buf1 to 6.4, 3.2, 1.6, 0.8, 0.4, 0.2, 0.1, 0.05, 0.025, and 0.0125 μM, and samples were injected in ascending order of concentration. All samples were injected for 120 s and dissociated for 60 s at a flow rate of 20 μL/min with the PBS running buffer.

Three curves were randomly selected from the results, and a smooth straight line was intercepted to determine the noise value of the instrument. The LOD was the corresponding concentration when the response was three times greater than the noise value.

### 2.6. Effect of Dissociation Time

In order to investigate the influence of dissociation time on the detection results, the dissociation times of 15, 30, 60, 120, and 240 s were tested. 5 mM CAP was diluted to 6.4, 3.2, 1.6, 0.8, 0.4, 0.2, 0.1, 0.05, 0.025, and 0.0125 μM with Buf1 for injection. The injection time was 120 s, and the flow rate was 20 μL/min.

### 2.7. Specificity of the SPR Sensor

5 mM CN, THP, FF, and CAP were, respectively, diluted to 6.4 μM with Buf1, and then they were injected successively. All samples were injected for 120 s and dissociated for 60 s at a flow rate of 20 μL/min.

### 2.8. Preparation and Detection of Milk Samples

Milk samples with a fat content of 4.4% (*w*/*w*) were obtained from a local market. Prior to analysis, milk samples were diluted 100 times with Buf1 and filtered through a polyvinylidene fluoride filter (0.45 μm). The filtered samples were centrifuged at 14000 rpm for 15 min, and the bottom layer was discarded. Then CAP was added to the milk samples to prepare 75, 20 nM (24.23, 6.46 ng/mL) samples. These samples and the control milk sample (without CAP added) were tested in triplicate. The flow rate was 20 μL/min. The injection time was 120 s, and the dissociation time was 60 s.

## 3. Results and Discussion

### 3.1. Design of Aptamer Immobilization Strategies

**Figure 1:**
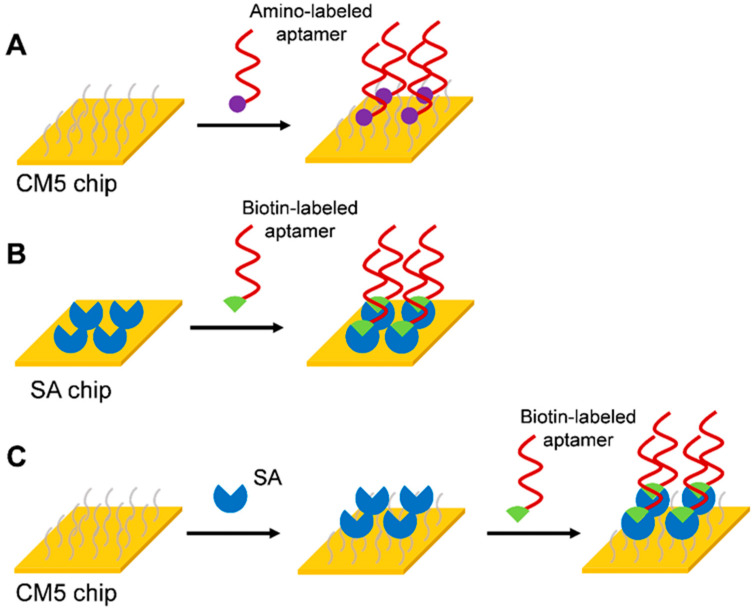
Immobilization strategies for aptamers. (**A**) Amino coupling strategy of CM5 chip, (**B**) Biotin-avidin coupling strategy of SA chip, (**C**) Biotin-avidin coupling strategy of CM5 chip.

Three strategies for immobilizing aptamers on the SPR sensor surface were designed: (1) Amino coupling strategy of CM5 chip (Figure 1A). The aptamer was immobilized on the CM5 chip surface by an amino coupling reaction by adding an amino label to the 5′ end of the aptamer. (2) Biotin-avidin coupling strategy of SA chip (Figure 1B). A biotin label was added to the 5′ end of the aptamer, and the SA on the surface of the SA chip was directly bound by the strong specific binding between SA and biotin. (3) Biotin-avidin coupling strategy of CM5 chip (Figure 1C). A high level of SA was immobilized on a CM5 chip by amino coupling reaction. Then the biotin-labeled aptamer was bound to SA. Due to the distance between the aptamer and the chip surface, which might affect its binding capacity to the corresponding target molecule, a triethylene glycol (TEG) linker was added to the 5′ end of the aptamer to increase the distance between the aptamer and the sensor surface.

### 3.2. Strategy of Amino Coupling on CM5 Chip

**Figure 2:**
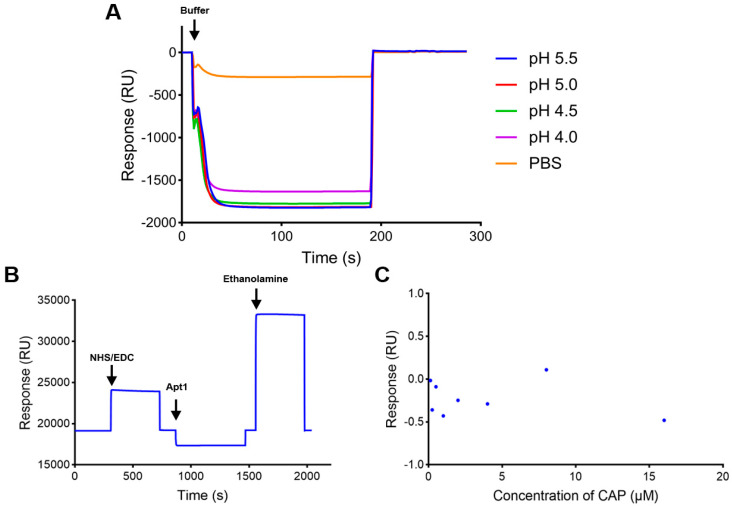
Characterization of the SPR biosensor using the CM5 chip with Apt1 immobilized. (**A**) pH scouting of Apt1 with different sodium acetate buffers. (**B**) Immobilization of Apt1 on the CM5 chip. (**C**) Activity of Apt1 on the CM5 chip.

The CM5 chip with the amino coupling method is commonly used in SPR biosensors. So, the aptamer recognition element was first prepared by an amino coupling reaction on a CM5 chip. Since there was no amino group in the CAP aptamer, an amino group was added to the 5′ end of the CAP aptamer to achieve direct binding on the CM5 chip. The calculated target immobilization level of Apt1 was about 5800 resonance units (RUs). Apt1 was immobilized on the CM5 chip after being diluted to 10 μM with sodium acetate buffer (pH 4.0) according to the result of pH scouting (Figure 2A). The immobilization level was 49 RUs (Figure 2B), which was lower than the target level. The low immobilization level was mainly due to the insufficient amino group in the aptamer structure, resulting in the inability to be immobilized on the chip surface in large quantities.

The activity of the aptamer biosensor was verified by injection of serial concentrations (0.125–16 μM) of CAP samples. As shown in Figure 2C, the response value generated by CAP samples was too low and had no correlation with the concentration magnitude. This may be due to the fact that the pH value of the aptamer solution was changed by sodium acetate buffer (pH 4.0) in the amino coupling process, which influenced its correct folding structure. Moreover, the amount of aptamers coupled on the chip surface was too small to produce a significant response for accurate detection. These results show that the strategy of directly fixing the aptamer to the chip surface by the amino coupling method could not realize the detection of CAP samples.

### 3.3. Strategy of Biotin-Avidin Coupling on SA Chip

**Figure 3:**
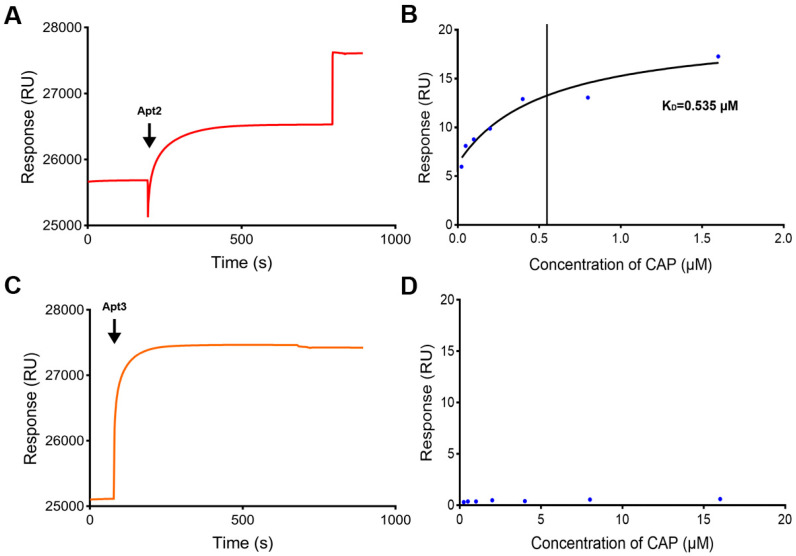
Characterization of the SPR biosensor using the SA chip. (**A**) Immobilization of Apt2 on the SA chip. (**B**) Fitting curves of CAP with Apt2 on the SA chip. (**C**) Immobilization of Apt3 on the SA chip. (**D**) Activity of Apt3 on the SA chip.

For ligands that were not suitable for amino coupling immobilization on the CM5 chip surface, they could be immobilized on the SA chip surface by a high-affinity biotin-avidin reaction. So, biotin-labeled aptamers were tried to be immobilized on an SA chip. The immobilization level of biotin-labeled aptamer (Apt2) on the SA chip surface was 1922 RUs (Figure 3A). After injection of CAP samples with a series of concentrations (0.025–1.6 μM), it was found that the binding process between the CAP samples and Apt2 was in a concentration-dependent manner (Figure 3B). The equilibrium dissociation constant (K_D_) was determined as 0.535 μM, indicating that the activity of the SA chip with Apt2 was good.

In order to know the influence of steric hindrance on the binding freedom between aptamers and target molecules, a TEG linker was added to the 5′ end of the aptamer (Apt3). TEG has a chemical formula of C_6_H_14_O_4_ and a linear structure that consists of three ethylene glycol units linked together by oxygen atoms with a hydroxyl group attached to each end, so the distance between the aptamer molecule and the chip surface would be increased. The immobilization level of Apt3 on the SA chip surface was 2314 RUs (Figure 3C). The binding activity was then tested by CAP samples. As shown in Figure 3D, the response value generated by CAP samples was extremely low and irregular. This result indicated that increasing the distance between the aptamer and the chip surface will result in a lower response value, so the SPR biosensor with Apt3 had poor activity. Therefore, adding a TEG linker could not enhance the response value, and increasing the density of aptamers on the chip would be the next choice.

### 3.4. Strategy of Biotin-Avidin Coupling on CM5 Chip

#### 3.4.1. Immobilization of SA on CM5 Chip

**Figure 4:**
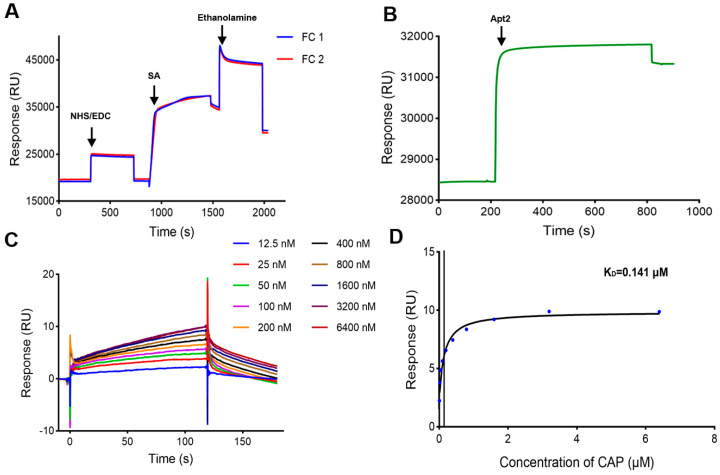
Characterization of the SPR biosensor using the CM5 chip with SA immobilized. (**A**) Immobilization of SA on the CM5 chip. (**B**) Immobilization of Apt2 on the SA immobilized chip. (**C**) Sensorgrams of CAP at different concentrations. (**D**) Fitting curve of CAP.

In order to increase the density of aptamers on the CM5 chip surface by the biotin-avidin coupling method, the number of SA immobilized on the chip surface needs to be increased. SA was immobilized on both FC1 and FC2 of a CM5 chip after being diluted to 100 μg/mL with sodium acetate buffer (pH 5.5). The immobilization level was 10807.6 RUs on FC1 and 9891.9 RUs on FC2 (Figure 4A).

#### 3.4.2. Immobilization of Apt2 on CM5 Chip and Activity of CM5 Chip

Apt2 was immobilized on FC2 by biotin–SA interactions, and the immobilization level was 2901.9 RUs (Figure 4B). CAP samples were injected and analyzed, and the response values increased in a concentration-dependent manner (Figure 4C,D), and the fitting curve was closer to the steady-state binding model compared to those from the SA chip (Figure 3B). The K_D_ was determined as 0.141 μM. These results showed that this CM5 chip with Apt2 had better activity than the SA chip with Apt2. Therefore, the strategy of biotin-avidin coupling on the CM5 chip was considered as the optimal method to immobilize the biotin-modified CAP aptamer on the SPR sensor surface and applied to determine CAP.

#### 3.4.3. LOD and Detection Range

The LOD was found to be 0.0016 ± 0.00029 μM (0.52 ± 0.09 ng/mL) by calculating instrument noise (3 × means of instrument noise), which was lower than the lowest concentration in the series of CAP samples. Since the binding of CAP to the aptamers reaches saturation at 3.2–6.4 μM (Figure 4D), the upper limit was determined as 3.2 μM. Therefore, the reliable detection range of CAP was determined to be 0.0125–3.2 μM (4.04 ng/mL–1.03 μg/mL).

### 3.5. Effect of Dissociation Time

**Figure 5:**
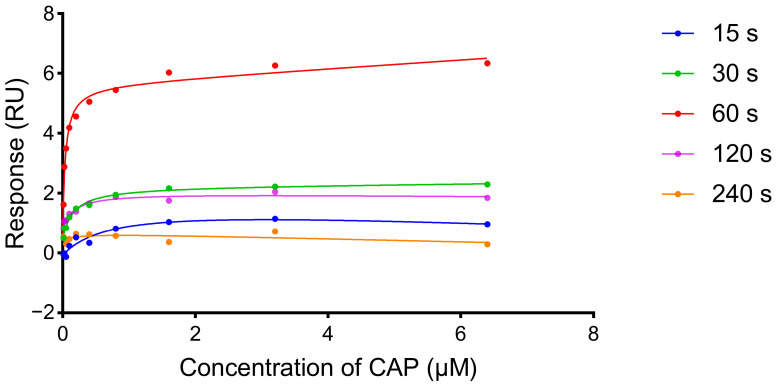
Influence of dissociation time on CAP detection. The dissociation time was 15 s, 30 s, 60 s, 120 s, and 240 s.

During the experiment, it was found that different dissociation times, which ensured enough time for the aptamer to restore its original spatial structure, would affect the response values. In order to determine the influence of the dissociation process, detection results of continuous injections with different dissociation times (15, 30, 60, 120, and 240 s) were investigated. PBS and Buf1 were selected as the running buffer and sample diluent, respectively, for all samples (Supplementary Material Figure S1). As shown in Figure 5, the response value gradually increased from 15 s to 60 s but decreased significantly from 60 s to 240 s, and the correlation with concentration was lost. The reason was considered that the spatial structure of the CAP aptamer was flexible, and the structure changed during the association and dissociation process. When the dissociation time was too long, the aptamer returned to the prebinding state, and it would take longer to recombine with CAP to reach the actual response value. When the dissociation time was too short, the CAPs and aptamers were not completely dissociated, and the binding sites became fewer, resulting in the reduction in response value. Therefore, 60 s was finally chosen as the optimal dissociation time in the CAP determination.

### 3.6. Specificity of the SPR Sensor

**Figure 6:**
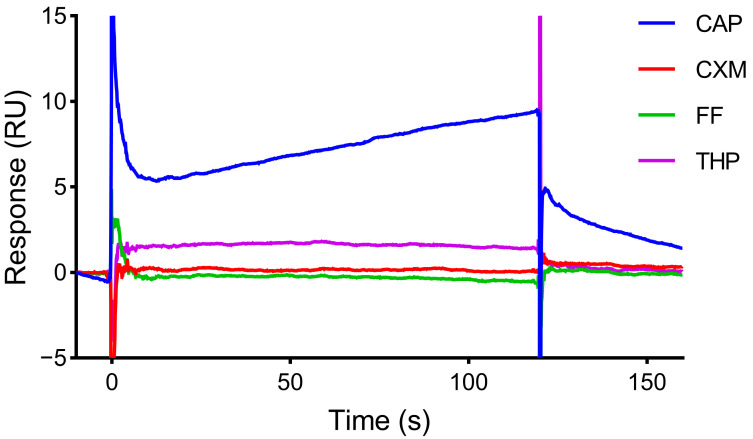
Specificity of CM5 chip with Apt2.

THP, FF, and CXM, which were similar in structure to CAP, were selected for specific evaluation. The same concentration of each compound was injected. As shown in Figure 6, the response values of THP, FF, and CXM were close to the baseline, and the response value of CAP was higher than those of the other three compounds. The results indicated that the aptamer-based SPR sensor had good specificity for CAP and can be used for CAP detection.

### 3.7. Robustness of the SPR Sensor

In order to determine whether this SPR sensor was feasible in a practical sample which has a more complex background, commercially available milk with relatively simple pretreatment was tested. The SPR biosensor based on the biotin-avidin coupling strategy of the CM5 chip was used to detect a series of CAP standard samples with the optimal scheme to build a standard curve prior to practical sample analysis. Then, milk was diluted, filtered, and centrifuged to remove the precipitate. Two different concentrations of CAP (20 and 75 nM in the final, i.e., 6.46 and 24.23 ng/mL) were added to the milk supernatant to prepare CAP-spiked samples. The SPR detection of these samples was performed, and the data were obtained by subtracting the response value of the blank milk sample from the response value of the CAP-added milk sample. The concentrations were calculated using the standard curve, and next the precision and accuracy were calculated.

As results shown in Table 3, the detected concentrations of the two samples were 18.59±3.91 nM and 57.77±9.28 nM, respectively, which were close to those of the CAP added. Furthermore, the precision was from 16% to 21%, and the accuracy was between 77% and 92%. These results have met the basic requirements of biological sample analysis. Nevertheless, the SPR sensor and the detecting conditions could be further optimized to obtain perfect results. It is worth noting that the samples we currently tested are milk diluted 100-fold, so the actual CAP concentration in the original milk sample should be multiplied by 100. In addition, if the CAP concentration in the sample is lower than the LOD, it may not be detected. Therefore, a positive result can be used to confirm the presence of CAP, while a negative result cannot be used to confirm the absence of CAP. In the future, we can reduce the dilution factor of milk samples by optimizing the pretreatment method of milk samples or optimize and improve the detection performance of the aptamer sensor, thereby lowering the LOD and achieving more sensitive detection. To sum up, the study preliminary demonstrates this SPR biosensor could be used to detect CAP in real samples such as milk.

## 4. Conclusions

In this study, a verified CAP aptamer was selected and used as a recognition element for the SPR biosensor to achieve the direct detection of CAP in a complex sample system. By optimizing the coupling strategy, running buffer, and dissociation time, the construction and application method of the aptamer-based SPR biosensor are systematically elucidated, which provides a reference for the research of other aptamer-based SPR biosensors. The results suggested that the optimal immobilization strategy of aptamers on the SPR sensor surface was indirect immobilization based on the CM5 chip. By using this method, the high immobilization level of biotin-labeled aptamer was achieved, which improved the stability and sensitivity of the aptamer-based SPR biosensor. Moreover, biotin reacted with SA without the addition of other buffers or reagents, avoiding the change in the folding structure of aptamers. After selecting the appropriate running buffer, sample diluent, and dissociation time, the SPR biosensor based on biotin-labeled aptamer had the specificity and could detect CAP in a real sample.

Although the SPR biosensor with the CAP aptamer as the recognition element has favorable specificity, there are still some issues in the accurate quantitative detection of CAP. Firstly, the binding of aptamers to their target molecules can be affected by several factors, such as the ionic composition and pH of buffers and the density of aptamers on the sensor surface. Second, the interaction of aptamers and their targets requires a long incubation time to form a stable complex, but samples in the SPR biosensor are flowing at a slow flow rate and cannot stay still absolutely for a long time. Third, the affinity of aptamers used in the SPR biosensor should be strong enough, at the nanomolar scale at least, to obtain the ideal stability and sensitivity. Thus, it is necessary to screen out aptamers with stronger affinity by SELEX and other novel technologies.

In view of the current study, despite aptamers having many advantages compared with antibodies in physicochemical properties, cost, and other aspects, it is more complicated to use them in quantitative detection. Aptamers have great application prospective in the SPR sensor field and so on for their many unique merits, as well as conducting further study to improve the performance of the sensor. The realization of aptamer-based SPR biosensors will expand the selection range of recognition elements to deal with more application scenarios and detection requirements in the food safety field.

## Figures and Tables

**Table 1:** Sequences of aptamers.

I.D.	Sequences
Apt1	5′-NH_2_-(CH_2_)_6_-ACT TCA GTG AGT TGT CCC ACG GTC GGC GAG TCG GTG GTA G-3′
Apt2	5′-Biotin-ACT TCA GTG AGT TGT CCC ACG GTC GGC GAG TCG GTG GTA G-3′
Apt3	5′-Biotin-TEG-ACT TCA GTG AGT TGT CCC ACG GTC GGC GAG TCG GTG GTA G-3′

**Table 2:** Composition of sample dilution buffers.

I.D.	Composition
PBS	137 mM NaCl, 2.7 mM KCl, 10 mM Na_2_HPO_4_, 2 mM KH_2_PO_4_
Buf1	100 mM NaCl, 20 mM Tris-HCl, 2 mM MgCl_2_, 5 mM KCl, 1 mM CaCl_2_, pH 7.4
Buf2	10 mM Tris-HCl, 40 mM MgCl_2_, pH 7.4
Buf3	100 mM NaCl, 20 mM Tris-HCl, 2 mM MgCl_2_, 5 mM KCl, 1 mM CaCl_2_, 0.02% Tween 20, pH 7.4

**Table 3:** Detection of CAP in milk samples (n = 3).

Nominal Concentration	Detected Concentration (mean ± SD, nM)	Precision (CV, %)	Accuracy (%)
20 nM (6.46 ng/mL)	18.59 ± 3.91	21	92
75 nM (24.23 ng/mL)	57.77 ± 9.28	16	77

## Data Availability

The original contributions presented in this study are included in the article. Further inquiries can be directed to the corresponding authors.

## Supplementary Materials

The following supporting information can be downloaded at https://www.mdpi.com/article/10.3390/bios15110706/s1, Figure S1: Responses of different running buffers and sample diluents.
