# Supplementary material for: Development of an Aptamer-Based Surface Plasmon Resonance Biosensor for Detecting Chloramphenicol in Milk
Source: Biosensors (Basel). 2025 Oct 22;15(11):706. doi: 10.3390/bios15110706 (PMC12650355; doi:10.3390/bios15110706)
Supplement: Supplementary file 1 [file biosensors-15-00706-s001.zip › biosensors-3881794-supplementary.pdf]

biosensors

MDPI

Supplementary Material

Development of an Aptamer-Based Surface Plasmon Resonance Biosensor for Detecting Chloramphenicol in Milk

Minyu Qi [1,†], Ningqi Xia [1,†], Xiying Wang [2], Xiaofei Wang [1], Hao Chen [1], Diya Lv [3,*] and Yan Cao [1,4,*]

[1] Department of Biochemical Pharmacy, College of Pharmacy, Naval Medical University, Shanghai 200433, China; jstzqi@163.com (M.Q.); xianingqiqi@163.com (N.X.)
[2] Suzhou Innovation Center of Shanghai University, Suzhou 215127, China
[3] Center for Instrumental Analysis, College of Pharmacy, Naval Medical University, Shanghai 200433, China
[4] Shanghai Key Laboratory for Pharmaceutical Metabolite Research, College of Pharmacy, Naval Medical University, Shanghai 200433, China
† These authors contributed equally to this work.
* Correspondence: lvdy2020@smmu.edu.cn (D.L.); caoyan@smmu.edu.cn (Y.C.)

1. Materials and Methods

1.1. Effect of Running Buffers and Sample Diluents

The SPR sensor for detecting CAP was prepared using the strategy of biotin-avidin coupling on the CM5 chip. In order to know the buffer influence on the binding of CAP and Apt2, four different groups of running buffers and sample diluents were set (running buffer/sample diluent): ⅰ) PBS/PBS, ⅱ) Buf1/Buf1, ⅲ) PBS/Buf2, ⅳ) PBS/Buf3. The concentrations of CAP samples were 6.4, 3.2, 1.6, 0.8, 0.4, 0.2, 0.1, 0.05, 0.025, and 0.0125 μM. All samples were injected for 120 s and dissociated for 60 s at a flow rate of 20 μL/min.

2. Results and Discussion

2.1. Effect of Running Buffers and Sample Diluents

CAP aptamers need to form and maintain the correct spatial structure under specific ion concentration conditions. Therefore, it was necessary to determine the optimal detection buffer for the SPR biosensor to ensure the quantitative detection performance. Four different groups of running buffers and sample diluents were set (running buffer/sample diluents): ⅰ) PBS/PBS, ⅱ) Buf1/Buf1, ⅲ) PBS/Buf2, ⅳ) PBS/Buf3. A series of CAP samples was analyzed under each condition, and the response values of each group were assessed.

As shown in Figure S1A-B, the response values of CAP in groups ⅰ and ⅱ were low, especially the response values in group ⅰ had no significant correlation with concentrations. The reason was conjectured that the aptamer cannot form and maintain the particular 3D structure to bind to the target molecule in the PBS buffer containing only Na^+ and K^+. In addition, the results of groups ⅲ and ⅳ (Figure S1C-D) indicated that Mg^{2+} alone or Tween 20 in the sample diluent was not applicable.

The above results were compared with the result of CAP samples when PBS was used as a running buffer and Buf1 was used as a sample diluent in 3.4.2. The detection results with the buffer condition of PBS/Buf1 were significantly better. Therefore, PBS was selected as the running buffer and Buf1 as the sample diluent.

 https://doi.org/10.3390/bios15110706

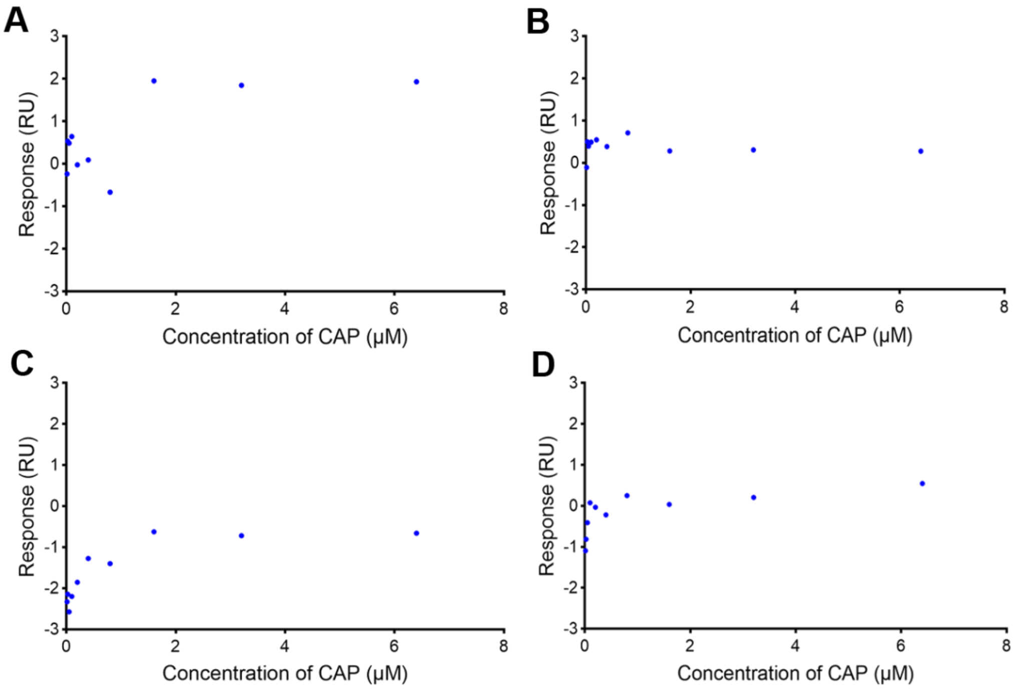

Figure S1. Responses of different running buffers and sample diluents. **(A)** PBS/PBS, **(B)** Buf1/Buf1, **(C)** PBS/Buf2, **(D)** PBS/Buf3.:.

Disclaimer/Publisher's Note: The statements, opinions and data contained in all publications are solely those of the individual author(s) and contributor(s) and not of MDPI and/or the editor(s). MDPI and/or the editor(s) disclaim responsibility for any injury to people or property resulting from any ideas, methods, instructions or products referred to in the content.
